# Case Report: Laser thermal ablation for severe primary hyperparathyroidism in a pregnant patient with MEN1 syndrome

**DOI:** 10.3389/fendo.2026.1964081

**Published:** 2026-10-12

**Authors:** Mateusz Obal, Marcin Motyka, Grzegorz Sokołowski, Tomasz Piskorz, Hubert Huras, Alicja Hubalewska-Dydejczyk

**Affiliations:** 1 Endocrinology, Oncology Endocrinology, Nuclear Medicine and Internal Medicine Clinical Department, University Hospital, Krakow, Poland; 2 Chair and Department of Endocrinology, Collegium Medicum, Jagiellonian University, Krakow, Poland; 3 Obstetrics and Perinatology Clinical Department, University Hospital, Krakow, Poland; 4 Department of Obstetrics and Perinatology, Collegium Medicum, Jagiellonian University, Krakow, Poland

**Keywords:** hypercalcemia, laser thermal ablation, MEN1, minimally invasive treatment, pregnancy, primary hyperparathyroidism

## Abstract

Primary hyperparathyroidism (PHPT) during pregnancy is a rare clinical entity associated with substantial maternal and fetal morbidity, particularly when complicated by severe hypercalcemia. We present the case of a 44-year-old multigravida at 27 + 5 weeks’ gestation who presented with symptomatic, severe hypercalcemia (3.12 mmol/L), and acute kidney injury. Diagnostic evaluation confirmed bilateral parathyroid lesions. Due to high surgical risk and patient refusal of traditional parathyroidectomy, ultrasound-guided laser thermal ablation (LTA) of the dominant right inferior adenoma was performed. The procedure induced a rapid reduction in parathyroid hormone (PTH) concentrations and normalization of serum calcium levels, accompanied by a marked recovery of renal function. Subsequent genetic testing identified a pathogenic MEN1 mutation, confirming multiple endocrine neoplasia type 1. At 39 weeks of gestation, the patient delivered a healthy infant via elective cesarean section, with no evidence of neonatal hypocalcemia. This case demonstrates that LTA may serve as a safe, effective, and minimally invasive alternative for managing severe PHPT in pregnancy when conventional surgery is contraindicated or declined. To our knowledge, this represents the first reported application of LTA in this specific clinical scenario.

## 1 Introduction

Primary hyperparathyroidism (PHPT) complicating pregnancy is an uncommon clinical condition. Although its exact incidence is not well-established, it is estimated to affect approximately 0.05% of women of reproductive age (1, 2). A solitary parathyroid adenoma accounts for roughly 85% of cases, whereas multiglandular disease or hyperplasia frequently associated with hereditary syndromes such as multiple endocrine neoplasia type 1 (MEN1), type 2 (MEN2), or hyperparathyroidism-jaw tumor (HPT-JT) syndrome is substantially less common (1, 3).

Hypercalcemia during pregnancy is typically mild; however, the risk of severe complications escalates dramatically when maternal serum calcium concentrations exceed 3.0 mmol/L. The most prevalent maternal complications include nephrolithiasis (24–36%) and acute kidney injury secondary to urolithiasis, gestational hypertension or preeclampsia (25%), and, less frequently, acute pancreatitis. Prior literature indicates that the risks of preterm delivery and spontaneous abortion are increased three- to fivefold in these patients. Regarding fetal complications, gestational hypercalcemia is most frequently associated with fetal growth restriction, amniotic fluid abnormalities (polyhydramnios or oligohydramnios), neonatal pulmonary hypertension, postpartum neonatal hypocalcemia, and an elevated risk of intrauterine fetal demise (2–4). Consequently, poorly controlled PHPT with profound hypercalcemia during gestation carries a serious prognosis, necessitating rigorous monitoring and intensive, often urgent, therapeutic intervention (2, 4).

## 2 Case presentation

A 44-year-old multigravida at 27 + 5 weeks’ of gestation was admitted to the Department of Obstetrics presenting with symptoms of left-sided renal colic and concurrent acute kidney injury. Upon presentation, the patient complained of nausea, vomiting, and severe left flank pain. Physical examination revealed marked left-sided costovertebral angle tenderness. At the time of admission, no abnormalities in fetal heart activity, preterm uterine contractions, or other signs suggestive of an increased risk of preterm delivery were observed. Patient’s medical history was notable for right renal atrophy secondary to nephrocalcinosis and a prior endoscopic calculus removal from the right pelvicalyceal system several years beforehand. No comprehensive metabolic evaluation for nephrolithiasis or endocrinological assessment had been performed prior to the current admission (for event timeline see **Figure 1**).

**Figure 1:**
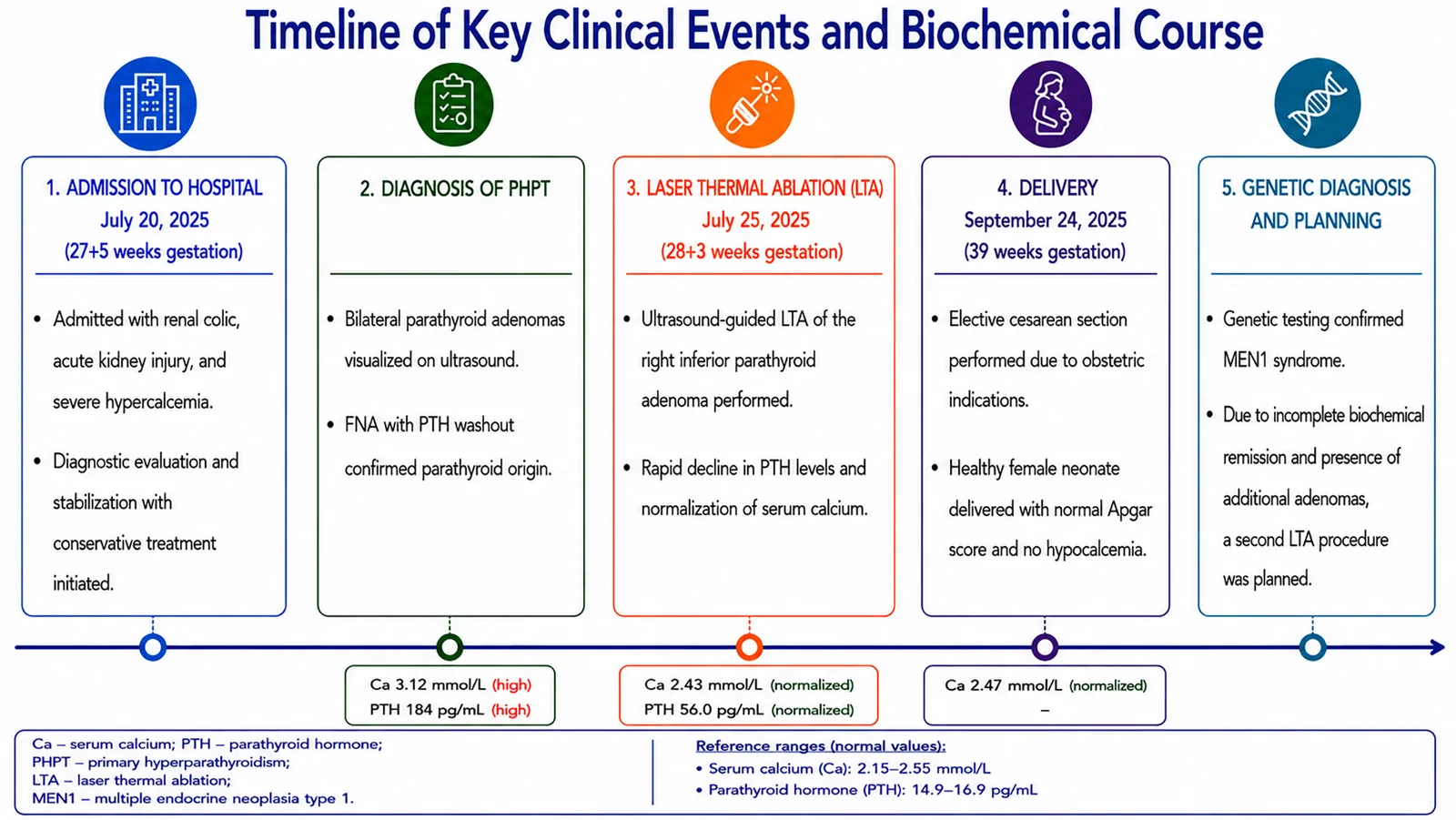
Timeline of key clinical events and biochemical course in a pregnant patient with primary hyperparathyroidism, including diagnosis, laser thermal ablation, delivery, and MEN1 diagnosis.

Initial laboratory investigations revealed severe hypercalcemia (corrected serum calcium: 3.12 mmol/L; reference range: 2.15–2.50), a severely reduced glomerular filtration rate (GFR) of 20 mL/min/1.73 m² (serum creatinine: 238.0 µmol/L; reference range: 44.0–80.0), a markedly elevated parathyroid hormone (PTH) level of 184.0 pg/mL (reference range: 14.9–56.9), and a serum phosphate concentration of 0.83 mmol/L (reference range: 0.81–1.45).

Urological ultrasonography demonstrated dilatation of the left pelvicalyceal system secondary to obstructive nephrolithiasis. Subsequent endocrinological ultrasound identified two hypoechoic, hypervascular lesions at the inferior poles of the thyroid gland, measuring 8 × 10 × 12 mm on the right and 7 × 7 × 9 mm on the left, highly suggestive of parathyroid adenomas (see **Figure 2**). Given the contraindication to parathyroid scintigraphy during pregnancy, FNA-PTH washout was performed to confirm the parathyroid origin of the lesions without exposing the patient or fetus to ionizing radiation. The PTH concentration in the washout fluid was 1042.0 pg/mL from the right lesion and 9.09 pg/mL from the left lesion, with the latter being confirmed as parathyroid tissue via cytological evaluation.

**Figure 2:**
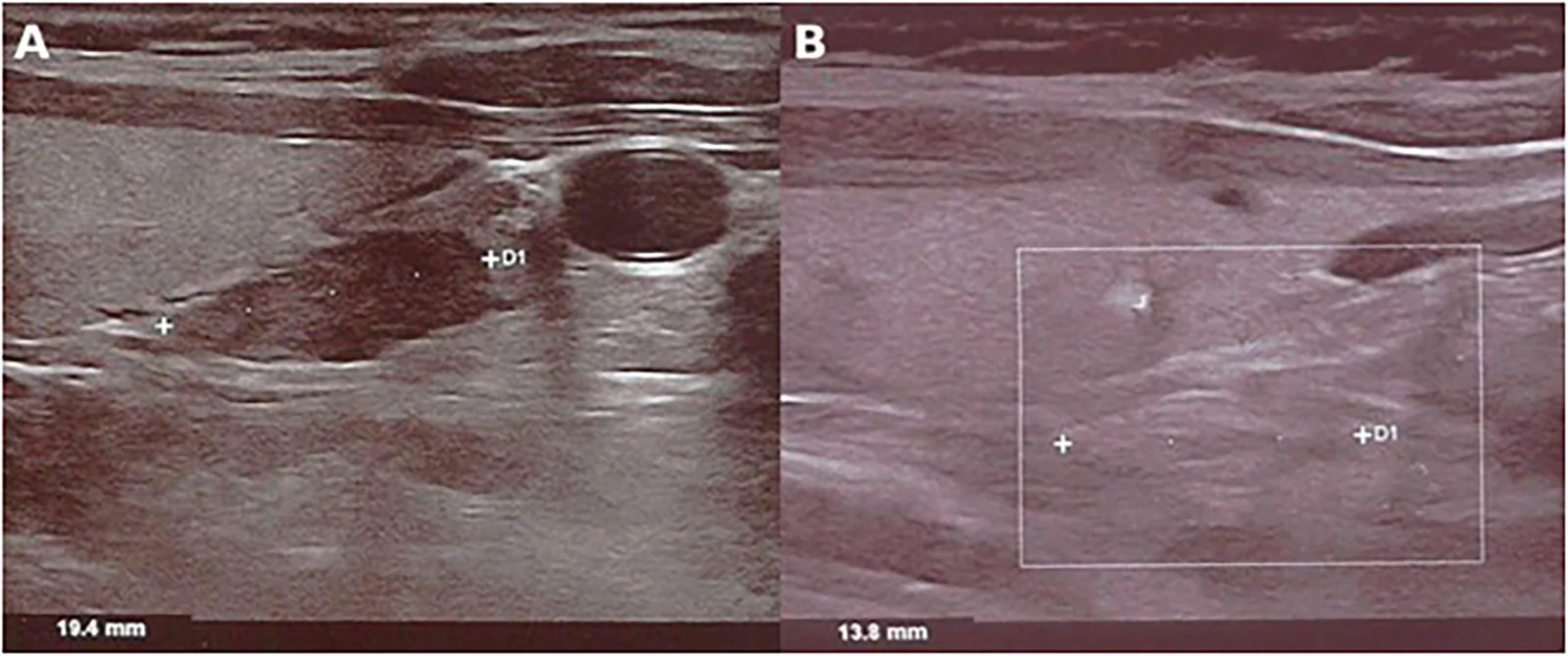
Ultrasound imaging of a right inferior parathyroid adenoma. **(A)** An oval, hypoechoic lesion located inferior to the lower pole of the right thyroid lobe, consistent with a parathyroid adenoma, prior to thermal ablation. **(B)** Follow-up ultrasound image obtained several months after thermal ablation; color Doppler imaging demonstrate the complete absence of internal vascularity.

A retrograde double-J ureteral stent was successfully placed, and the patient was initiated on intensive intravenous hydration along with empirical antibiotic therapy (ceftriaxone). Despite aggressive hydration and the administration of loop diuretics, maternal serum calcium levels remained critically elevated. Given the substantial risk of imminent pregnancy loss, surgical intervention was recommended; however, the patient firmly declined conventional parathyroidectomy. As an alternative, ultrasound-guided laser thermal ablation (LTA) of the right inferior adenoma was proposed and subsequently approved by the Bioethics Committee of the Regional Medical Chamber in Krakow.

On July 25, 2025, the patient underwent LTA of the right inferior parathyroid adenoma, with a total delivered energy of 3950.6 J. The procedure was uneventful, and post-procedural obstetric evaluation confirmed stable fetal well-being. Immediately following the intervention, the serum PTH concentration decreased from a pre-procedural value of 163 pg/mL to 56 pg/mL. Normalization of serum calcium levels was achieved within the subsequent days. Furthermore, renal function demonstrated a remarkable recovery, with the GFR improving significantly to 90 mL/min/1.73 m². Following LTA, no additional calcium-lowering pharmacotherapy was required, and loop diuretics were discontinued. The patient was advised to maintain adequate oral hydration throughout the remainder of pregnancy. During follow-up, serum PTH levels remained mildly elevated but were substantially lower than baseline values (**Table 1**), while serum creatinine levels remained within the reference range. A single transient episode of hypercalcemia (2.74 mmol/L) occurred during outpatient follow-up and was attributed to inadequate oral hydration; normocalcemia was promptly restored after increasing oral fluid intake. No procedure-related complications were observed either immediately after LTA or during follow-up.

**Table 1:** Biochemical course before and after laser thermal ablation.

Date and time point	Parathyroid hormone (pg/ml) reference range: 14.90–56.90	Calcium (mmol/l) reference range: 2.15–2.50	Phosphorus (mmol/l) reference range: 0.81–1.45
20.07.2025 (Admission)	184.0	3.12	0.83
25.07.2025 (Before ablation)	163.0	2.91	0.57
25.07.2025 (After ablation)	56.0	2.84	0.50
26.07.2025	—	2.44	0.65
28.07.2025	77.0	2.43	0.75
30.07.2025	66.0	2.50	0.67
13.08.2025	96.0	2.74	0.72
03.09.2025	—	2.47	0.77

Due to the bilateral nature of the parathyroid lesions, genetic screening was performed, identifying a pathogenic heterozygous variant in the MEN1 gene (c.654 + 1G>A), which confirmed the diagnosis of MEN1 syndrome. On September 24, 2025, at 39 weeks of gestation, the pregnancy was successfully concluded via an elective cesarean section. Both the surgical procedure and the postpartum course were unremarkable and free of complications. A healthy female infant was delivered with an Apgar score of 10. The neonate showed no clinical or laboratory evidence of postpartum hypocalcemia, which represents a significant risk in pregnancies complicated by maternal hypercalcemia. Following successful delivery, the patient was scheduled for a secondary LTA of the contralateral left inferior parathyroid adenoma. Moreover, following confirmation of the MEN1 gene mutation, the patient was referred for further evaluation of other MEN1-associated manifestations, including pituitary MRI and CT imaging of the chest, abdomen, and pelvis. Genetic counseling was also recommended for the patient’s family, with cascade genetic testing offered to at-risk relatives.

## 3 Discussion

The management of mild PHPT during pregnancy typically relies on conservative measures, primarily adequate oral or intravenous hydration. In more severe or refractory cases, pharmacological interventions such as loop diuretics or cinacalcet may be cautiously employed, provided that a stringent risk-benefit analysis justifies their use. Conversely, the administration of salmon calcitonin and denosumab should generally be avoided due to a lack of robust safety data in the pregnant population. Similarly, bisphosphonates are not recommended during gestation given the potential risk of impaired fetal skeletal development (1, 2, 4, 5).

In cases of symptomatic or severe hypercalcemia secondary to PHPT, surgical resection of the hyperfunctioning parathyroid tissue remains the definitive causal treatment of choice, with the second trimester representing the optimal window for surgical intervention (1, 2, 4, 5). However, when conventional surgery carries prohibitive risks or the patient declines operative management, minimally invasive causal alternatives must be explored.

Image-guided thermal ablation has emerged as a viable, minimally invasive technique that utilizes thermal energy to induce localized coagulative tissue necrosis. Currently, four primary modalities are recognized in clinical practice: radiofrequency ablation (RFA), laser thermal ablation (LTA), high-intensity focused ultrasound (HIFU), and microwave ablation (MWA) (6). LTA for primary hyperparathyroidism has been shown to represent an effective and safe therapeutic approach, capable of achieving disease remission or significant improvement in calcium–phosphate homeostasis (7, 8).

In patients with MEN1-associated primary hyperparathyroidism subtotal parathyroidectomy representing the preferred initial approach in most cases. However, thermal ablation may be considered a potential alternative in selected patients who, after being fully informed of the benefits and limitations of both approaches, decline surgical treatment or are not suitable surgical candidates. Thermal ablation may be associated with a higher risk of persistent or recurrent disease compared with surgery, particularly given the multiglandular and potentially asynchronous nature of MEN1-associated PHPT. Although ablation can be repeated if necessary, both repeat ablation and subsequent surgery may be technically more challenging due to post-procedural fibrosis. Therefore, long-term biochemical and imaging follow-up is essential.

In the present case, LTA (EchoLaser) was utilized, employing light energy at a wavelength of 1064 nm as the heat source. This energy is delivered directly into the target lesion via a 0.3-mm quartz optical fiber introduced through a fine 21-gauge (0.8-mm) needle. A critical prerequisite of this technique is the requirement for continuous, real-time ultrasound guidance to ensure geometric precision (6). The total energy delivered is titrated based on the volume of the target lesion. In the context of parathyroid interventions, the primary objective is the complete destruction of the hyperfunctioning tissue, which is confirmed post-procedurally by the absence of internal vascularization on color Doppler imaging and a significant reduction in serum PTH levels relative to baseline (8). Given the close anatomical proximity of the parathyroid glands to vital cervical structures including the trachea, esophagus, and major vessels the implementation of hydrodissection is imperative. This maneuver involves the ultrasound-guided injection of a fluid barrier (typically dextrose or saline) to create a physical buffer between the adenoma and adjacent structures, thereby mitigating the risk of thermal injury.

LTA has a favorable safety profile and is an established treatment modality for benign thyroid nodules, with pregnancy not considered an absolute contraindication to its use (6). However, evidence regarding parathyroid thermal ablation during pregnancy remains very limited and is based predominantly on individual case reports. Although isolated cases involving other thermal ablation techniques, including microwave ablation (MWA), have been reported in pregnant patients with primary hyperparathyroidism (9, 10), to the best of our knowledge, the present case represents the first reported use of LTA for the treatment of primary hyperparathyroidism during pregnancy. This observation suggests that pregnancy itself may not preclude the use of LTA in carefully selected patients; however, an individualized assessment of potential risks and benefits remains essential.

In this case, given the presence of two parathyroid adenomas, complete normalization of PTH levels following ablation of a single lesion was considered unlikely. As the patient declined surgical treatment, the primary therapeutic goal was to reduce serum calcium levels and achieve adequate control of hypercalcemia, thereby reducing the risk of hypercalcemia-related maternal and fetal complications. Following LTA, sustained biochemical control was achieved, allowing the pregnancy to progress to delivery without significant recurrent hypercalcemia or pregnancy-related complications and without the need for additional calcium-lowering pharmacotherapy.

## 4 Conclusions

In patients with primary hyperparathyroidism, surgery remains the standard first-line treatment. Laser thermal ablation (LTA) may represent an individualized, minimally invasive therapeutic option for carefully selected patients who are unsuitable candidates for surgery or decline surgical intervention. In MEN1-associated primary hyperparathyroidism, the multiglandular nature of the disease and the high risk of recurrence make subtotal parathyroidectomy the standard surgical approach. Therefore, the use of LTA in these patients requires careful consideration, particularly given the potential for post-ablation fibrosis to complicate subsequent surgical intervention. In the present case, LTA provided rapid biochemical control, with normalization of serum calcium levels and recovery of renal function, thereby potentially reducing the risk of maternal and fetal complications associated with severe hypercalcemia. Importantly, this case suggests that LTA may have the potential to achieve biochemical control of hypercalcemia associated with primary hyperparathyroidism during pregnancy when conventional surgical management is declined. Nevertheless, given the limited evidence regarding the use of LTA in MEN1-associated primary hyperparathyroidism, particularly during pregnancy, further studies with long-term follow-up are needed to establish its efficacy, safety, and appropriate role in this unique clinical setting.

## Data Availability

The original contributions presented in the study are included in the article/supplementary material. Further inquiries can be directed to the corresponding author.
